# Detection Methods of COVID-19

**DOI:** 10.1177/2472630320962002

**Published:** 2020-09-30

**Authors:** Amira Echtioui, Wassim Zouch, Mohamed Ghorbel, Chokri Mhiri, Habib Hamam

**Affiliations:** 1 ATMS Lab, Advanced Technologies for Medicine and Signals, ENIS, Sfax University, Sfax, Tunisia; 2 King Abdulaziz University (KAU), Jeddah, Saudi Arabia; 3 Department of Neurology, Habib Bourguiba University Hospital, Sfax, Tunisia; 4 Neuroscience Laboratory “LR-12-SP-19,” Faculty of Medicine, Sfax University, Sfax, Tunisia; 5 Faculty of Engineering, Moncton University, Moncton, NB, Canada

**Keywords:** COVID-19, convolutional neural network, CNN, deep learning, diagnosis

## Abstract

Since being first detected in China, coronavirus disease 2019 (COVID-19) has spread rapidly across the world, triggering a global pandemic with no viable cure in sight. As a result, national responses have focused on the effective minimization of the spread. Border control measures and travel restrictions have been implemented in a number of countries to limit the import and export of the virus. The detection of COVID-19 is a key task for physicians. The erroneous results of early laboratory tests and their delays led researchers to focus on different options. Information obtained from computed tomography (CT) and radiological images is important for clinical diagnosis. Therefore, it is worth developing a rapid method of detection of viral diseases through the analysis of radiographic images. We propose a novel method of detection of COVID-19. The purpose is to provide clinical decision support to healthcare workers and researchers. The article is to support researchers working on early detection of COVID-19 as well as similar viral diseases.

## Introduction

Coronavirus disease 2019 (COVID-19) is an infectious disease caused by the severe acute respiratory syndrome coronavirus 2 (SARS-CoV-2), which was first detected in December 2019 in Wuhan, China, and has since rapidly spread to nearly every country.^1^ Currently, very little is known about the virus’ mutation patterns, the possibility of reinfection, and possible long-term health effects; and, while fatal, it remains too early to pinpoint the mortality rate for each patient demographic, given the political and medical issues surrounding the reporting and collection of data.^1^ When facing the unknown, prevention and early treatment procedures for infected patients comprise the most viable and ethical attitude. To date (September 16, 2020), there are 29,930,994 confirmed cases globally with 942,667 deaths and 21,688,376 recovered patients.^1^

It is still debatable whether the COVID-19 virus was transferred to humans through animals before human-to-human transmission.^2,3^ Symptoms of this disease include a headache, sore throat, cough, fever, and loss of smell or taste.^3^ The disease is caused by SARS-CoV-2 and asseverated to be transmitted from person to person by multiple means, including aerosols, fomites, and droplets.^4^

Fever, cough, and shortness of breath are the most important symptoms in infected individuals for the diagnosis of COVID-19. These symptoms may show carrier characteristics by not being seen in infected individuals. Pathological tests performed in laboratories are taking more time. A fast and accurate diagnosis is necessary for an effective struggle against COVID-19. For this reason, several experts started to use radiological imaging methods. These procedures are performed with computed tomography (CT) or X-ray imaging techniques. COVID-19 cases have similar features in CT images in the early and late stages. It shows a circular and inward diffusion from within the image.^5^ Therefore, radiological imaging provides the detection of suspicious cases with an accuracy of 90%. Moreover, as deep learning continues to gain ground in medical procedures and techniques for diagnostic purposes, COVID-19 imaging testing could largely benefit from this strong nonlinear modeling capability.

The convolutional neural network (CNN)^6^ is a powerful tool that is widely used for image classification. Its hierarchical structure and efficient feature extraction from an image make CNN a dynamic model for image classification. Initially, layers are organized in three dimensions: depth, height, and width. The neurons in a given layer do not attach to all the neurons in the next layer, but only to a limited number of neurons in that layer. Finally, an output is reduced to a single probability vector score, coordinated with the depth dimension.

The CNN classifier uses various layers—convolution layer, pooling layer, flatten layer, and fully connected layer—for model-building and testing purposes. The CNN model uses these steps:

- Feature extraction: Several convolutions and pooling operations are used to evaluate and monitor potential features. The Maxpooling layer is used to reduce the spatial size of the convolved features.
- Classification: In this step, the fully connected layers act as a classifier. It uses the extracted features and evaluates the probability for the object in the image.

The main objective of this work is to propose a new CNN-based method to detect COVID-19 among tested subjects. The main research contributions of this article are the following:

- We propose a new method to detect COVID-19 among tested subjects using radiographic images.
- We carefully analyze radiographic images of patients’ lungs to detect COVID-19.
- The results obtained are evaluated using these performance criteria: accuracy, precision, recall and F1 score.
- The proposed method allows the detection of patients with COVID-19 with an accuracy of 91.34%.

The originality of this work lies in the following aspects: First, we developed a CNN-based model that consists of 10 convolutional layers followed by batch normalization, Maxpooling, a SoftMax layer, and three fully connected layers; second, we looped over all layers in the proposed model and trained them so that we could efficiently detect the virus from the X-ray images; and, third, our method serves as a reliable clinical decision support.

The rest of the article is organized as follows. In the next section, we will provide an overview of related works. Materials and Methods will describe the proposed approach in detail; additional materials and methods, as well as the results, are presented and discussed in the Performance Criteria and Discussion sections. Finally, we conclude our study in the last section.

## Related Works

Recently, the effectiveness of chest CT scans in the detection of COVID-19 among potential patients has caught the attention of researchers. We review studies on the use of radiographic images to aid in the diagnosis of COVID-19. In Ref.^7^, the authors have developed a predictive model to distinguish COVID-19 pneumonia from influenza A viral pneumonia using extensive learning techniques. The CNN model was used for such predictions. The maximum accuracy obtained from the prediction model was 86.7%.

In Ref.^8^, the authors have developed an artificial intelligence–based CT analysis tool for the detection and quantification of COVID-19. The system automatically extracted slices of opacities in the lungs. The developed system achieved a sensitivity of 98.2% and a specificity of 92.2%. The result of the system provides a quantitative measure of opacity and a 3D display of the volume of opacities. The system is robust with respect to pixel spacing and slice thickness.^8^

In Ref.^9^, the authors developed a deep learning model called COVNet to extract visual characteristics from chest CT scans to detect COVID-19. They used the visual characteristics to distinguish community-acquired pneumonia from other lung diseases other than pneumonia. COVNet is not, however, able to classify the severity of this disease. In Ref.^10^, the authors studied a dataset they called COVIDx and COVID-Net designed to detect COVID-19 from chest X-ray images. The dataset consists of chest X-ray images in four classes: radiographs of non-infected cases, bacterial radiographs, viral radiographs for COVID-19, and non-COVID-19 pneumonia-positive radiographs. They reported an overall accuracy of 83.5%. The lowest positive predictive value was reported for the non-COVID-19 class (67.0%), and the highest for the normal class (95.1%). To improve on previous studies, in Ref.^11^, the authors presented another CNN with fewer parameters but a superior performance. The authors used the same dataset as in Ref.^10^ to create COVID-ResNet to differentiate COVID-19 cases from the other four pneumonia cases and achieve a better performance than with COVID-Net.

Xu et al.^11^ developed a predictive method to distinguish COVID-19 pneumonia from influenza A viral pneumonia using extensive learning techniques and the CNN model. The maximum accuracy obtained from the prediction model was 86.7%. Shan et al.^12^ developed a deep learning–based system called VB-net for automatic segmentation of all lung and infection sites using chest CT. Narin et al.^13^ proposed an automatic transfer model based on CNN for the prediction of COVID-19 in chest X-ray images. They used the InceptionV3, Inception-ResNetV2, and ResNet50 models for improved predictions. The pre-formed ResNet50 model produced an accuracy of 98%, which is better than in Refs.^12,14^. Wang et al.^14^ studied radiographic changes in CT images of infected patients. They developed a prediction model based on depth learning using the modified learning transfer technique. Features are extracted from the CT images for prior diagnosis. The accuracy rate—89.5%—obtained by this method is better than that of the model in Ref.^12^ and is more time efficient.

Ali et al.^15^ constructed a CNN based on the InceptionV2, Inception-ResNetV3, and ResNet50 models for the classification of COVID-19 chest X-rays into COVID-19 and normal classes. They found a good correlation between CT image results and the PCR approach.^16^ In Ref.^17^, the CNN architectures were adopted on 224 COVID-19 images, 700 non-COVID-19 pneumonia images, and 504 normal cases, for which they reported an overall accuracy of 97.82%. In Ref.^11^, the authors used CT images to predict COVID-19 cases, in which they deployed the Inception transfer and learning model to establish an accuracy of 89.5%, with a sensitivity of 87.0% and a specificity of 88.0%.

## Materials and Methods

**Figure 1.**
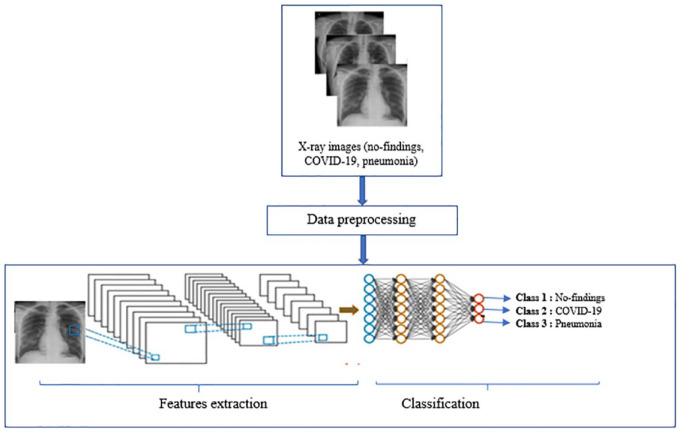
Flow diagram of the proposed method.

In this study, we propose a new CNN-based method of classifying COVID-19, pneumonia, and no-findings chest X-ray images. **Figure 1** shows the flow diagram of the proposed method.

### X-Ray Image Dataset

In this work, we use 500 chest X-ray images of COVID-19 obtained from the open-source GitHub repository, shared by Dr. Joseph Cohen,^18^ and the Covid-19 Radiography dataset.^19^ We also used the ChestX-ray8 database^20^ on no-findings and pneumonia images.

**Figure 2.**
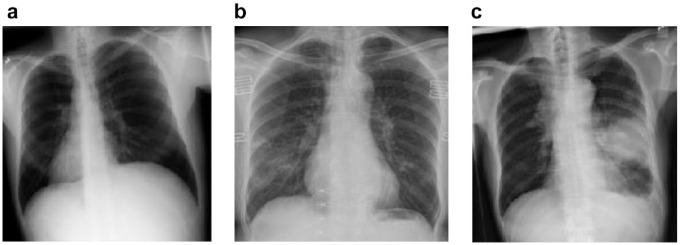
The sample X-ray images used in the experimental analysis of this work: (a) chest X-ray image of normal patient (no-findings), (b) chest X-ray image of coronavirus disease 2019 (COVID-19) patient, and (c) chest X-ray image of pneumonia patient.

**Figure 2** shows some examples of chest X-ray images from the prepared dataset.

**Table 1** shows the summary of the dataset we used, which was split into three folders: no-findings, COVID-19, and pneumonia.

**Table 1.** Summary of the Dataset Used.

Classes	Number of Images
No-findings	500
COVID-19	500
Pneumonia	500

COVID-19, coronavirus disease 2019.

### Proposed Model

**Figure 3.**
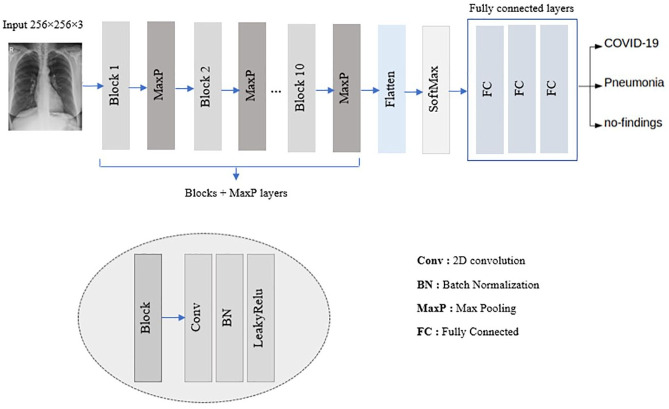
The proposed convolutional neural network (CNN) architecture.

**Figure 3** shows the proposed CNN architecture used to classify the chest X-ray images.

To detect COVID-19 cases, we have created a very simple CNN model composed of 10 convolutional layers followed by batch normalization, Maxpooling, a SoftMax layer, and three fully connected layers. The convolution layers have 32 filters, each of which has a kernel size of 3×3.

We have used a batch normalization operation to normalize the input; this operation has other advantages, notably reducing the training time and increasing the stability of the model. The activation function used was LeakyReLU, which is a variant of the ReLU operation used to prevent neuron death.

The Maxpooling method is used in all pooling operations. It reduces an input by taking the maximum of a region determined by its filter. The neurons of this layer are connected to all the activation functions of the previous layer. The main responsibility of the Maxpooling layer is to classify the convolutional features extracted from the image datasets into the defined classes.

The Softmax layer is simply used to interpret the probability values of the results of the activation functions of the previous layer. In cases of diagnosed disease, the values can be interpreted in three classes.

Finally, the three fully connected layers act as a classifier. They use extracted features and evaluate the probability of an object’s presence in the image. Usually, activation functions and a dropout layer are used to establish nonlinearity and minimize overfitting, respectively.

## Performance Criteria

A variety of performance criteria can be used to evaluate the performance of classification models, namely classification accuracy, sensitivity, specificity, precision, recall, and F1 score.

Sensitivity and specificity are two suitable criteria that can be used to report model performance. These criteria are also widely used in the medical field.

### Definition of the Terms

The cross-validation estimator was used and resulted in a confusion matrix, as shown in **Table 2**. The confusion matrix has the following four expected results:

**Table 2.** Confusion Matrix.

	Predicted Positive	Predicted Negative
Actual Positive	TP	FN
Actual Negative	FP	TN

FN, false negative; FP, false positive; TN, true negative; TP, true positive.

1. *TP* (true positive) is a number of anomalies and was identified with the correct diagnosis.
2. TN (true negative) is an incorrectly measured number of regular instances.
3. FP (false positive) is a Type 1 error, a set of regular instances that are classified as an abnormality diagnosis.
4. FN (false negative) is a Type 2 error, a list of abnormalities observed as an ordinary diagnosis.

In what follows, we present the performance criteria adopted to assess the performance of the different pretrained model used.

- *Accuracy* is defined by the rate of correctly classified images.

(1) Accuracy=TP+TN/(TP+TN+FP+FN)

- *Precision* is used to give relationship between the TP predicted values and FP predicted values.

(2) Precision=TP/(TP+FP)

- *Recall* is defined as the ratio of the total number of correctly classified positive patients to the total number of positive patients. It should be as high as possible.

(3) Recall=TP/(FN+TP)

- *F1 score*: It is difficult to compare two models with low precision and high recall, or vice versa. Thus, to render them comparable, we use the F1 score. It allows for the measurement of the Recall and Precision values at the same time. It uses the harmonic mean in place of the arithmetic mean by penalizing the extreme values more.

(4) F1score=2*(precision*recall)/(precision*recall)

### Results

The input image size of the proposed model was set to 256×256 pixels. Adam with a learning rate of 0.0003 was used as an optimizer, and the loss function was the cross-entropy loss. We trained the network by using a batch size of 32 and 200 training epochs.

In this study, we propose a CNN model to detect COVID-19 cases in chest X-ray images. The proposed model was tested on 225 images and achieved an average accuracy of 91.34% in classifying COVID-19-positive, pneumonia, and no-findings images. The classification accuracies are 94.14%, 90.97%, and 88.92% for the categories of COVID-19-positive, pneumonia, and no-findings, respectively.

**Table 3** shows the accuracy, recall, F1 score, and precision of the proposed model. It demonstrates that our model classified the COVID-19 and no-findings classes slightly better than the pneumonia class in terms of all the performance criteria. The classification of COVID-19 yields better results than the no-findings class with respect to accuracy, precision, and F1 score. The classification of no-findings is, however, superior to that of COVID-19 in terms of recall. Overall, the CNN model classified COVID-19 slightly better than the pneumonia and no-findings classes.

**Table 3.** Classification Report for the Proposed Model.

Class	Precision	Recall	F1 Score	Accuracy
COVID-19	96.00%	86.00%	91.00%	94.14%
No-findings	89.00%	94.00%	91.00%	90.97%
Pneumonia	88.00%	85.00%	87.00%	88.92%
Average	91.00%	88.33%	89.66%	91.34%

COVID-19, coronavirus disease 2019.

**Figure 4.**
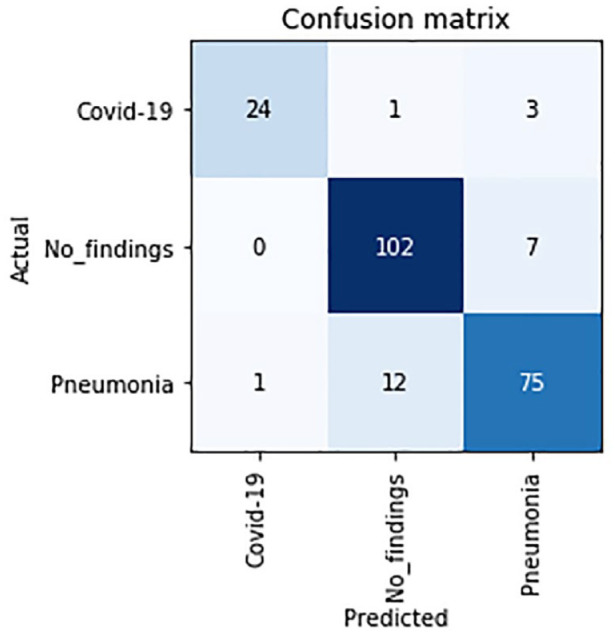
Confusion matrix of the proposed model.

The associated confusion matrix is shown in **Figure 4**.

## Discussion

Our results show a high value of the recall rate of the COVID-19 classification. It is worthwhile to note that this result is desirable, because a high recall value translates to a low number of false-negative cases. This is important because minimizing COVID-19 false-negative cases is in line with the main goal of this study—to better contribute to the clinical decision support.

It can be observed that our proposed model can achieve good precision for COVID-19 cases (96%), allowing us to limit the number of missed COVID-19 cases as much as possible.

We consider in **Table 4** a summary of the findings on automatic diagnosis of COVID-19 based on chest X-ray images and a comparison with our proposed model. The summary proves that our proposed model outperforms the other models with respect to accuracy.

**Table 4.** Summary of the Research on Automatic Diagnosis of COVID-19 Based on Chest X-Ray Images.

References	Image Type	Number of Cases	Model Used	Accuracy
^21^	Chest CT	224 Viral pneumonia 219 COVID-19 (+) 175 Healthy	Location Attention + ResNet	86.7%
^22^	Chest X-ray	125 COVID-19 (+) 500 No-findings 500 Pneumonia	DarkCovidNet	87.02%
Proposed model	Chest X-ray	500 COVID-19 (+) 500 No-findings 500 Pneumonia	Proposed CNN model	91.34%

CNN, convolutional neural network; COVID-19, coronavirus disease 2019; CT, computed tomography.

In Ref.^21^, the authors developed a new method to automatically screen for COVID-19 by using deep learning techniques. They showed that models with the location attention mechanism have the capability to classify COVID-19 on chest X-rays in a precise way, achieving an overall accuracy rate of around 86.7%. Therefore, these models may present promising diagnostic alternatives for clinicians.

The number of model samples used is, however, limited. This requires that the training and testing of samples should be expanded to enhance the accuracy in the future. In addition, the general performance of their method^21^ should be verified with a large dataset.

Tulin et al.^22^ suggest the DarkCovidNet model for automatically detecting and classifying COVID-19 cases from X-ray images. It is based on an end-to-end architecture that does not use feature extraction methods. It needs raw chest X-ray images to return the diagnosis. This model is capable of performing tasks with an accuracy of 87.02%.

In addition, it can be used to help radiologists invalidate their initial screening and can be used via the cloud to immediately screen patients. The limitation of DarkCovidNet lies in the use of a limited number of COVID-19 X-ray images. Most of these earlier studies, however, give little data to develop the model.

The model has been trained and tested on a small dataset of a few hundred chest X-ray images of various pneumonia cases, COVID-19 cases, and no-findings from different publicly available datasets.

Our model is less demanding in terms of computational efforts than other pretrained models and has yielded promising results. The performance can be improved further once more training data become available. Despite promising results, our proposed model still needs clinical research and testing, but with its higher accuracy and precision in detecting COVID-19 cases, our model can play a greater role in helping radiologists and health experts intervene.

## Conclusion

In this work, we introduced a simple but effective CNN model for the detection of COVID-19 disease from chest X-ray images. Although we achieved a fairly high detection accuracy, precision, and recall of COVID-19, this does not mean that it is a production-ready solution, especially with the limited number of images currently available. The objective of this study is to provide radiologists, scientists, and the research community with a simple CNN model that can be adopted for the early diagnosis of COVID-19 and hopefully serve as a basis for accelerating research in this direction.
